# Long travel times from health center to hospital reduce caesarean section access: a study from Kirehe District, Rwanda

**DOI:** 10.11604/pamj.2023.46.30.25504

**Published:** 2023-09-20

**Authors:** Hillary Miller, Niclas Rudolfson, Theoneste Nkurunziza, Teena Cherian, Daniella Kayitesi, Christian Mazimpaka, Fredrick Kateera, Robert Riviello, Bethany Hedt-Gauthier

**Affiliations:** 1 Department of Biostatistics, Harvard TH Chan School of Public Health, Boston, United States of America,; 2 Department of Global Health and Social Medicine, Harvard Medical School, Boston, United States of America,; 3 Word Health Organization Collaborating Center for Surgery and Public Health, Department of Clinical Sciences Lund University, Lund, Sweden,; 4 Program in Global Surgery and Social Change, Harvard Medical School, Boston, United States of America,; 5 Partners In Health/Inshuti Mu Buzima, Kigali, Rwanda,; 6 Department for Sport and Health Sciences, Technical University of Munich, Munich, Germany,; 7 Center for Surgery and Public Health, Brigham and Women´s Hospital, Boston, United States of America

**Keywords:** C-section, global surgery, decentralization, Africa

## Abstract

**Introduction:**

timely access to safe cesarean section (c-section) delivery can save the lives of mothers and neonates. This paper explores how distance affects c-section access in rural sub-Saharan Africa, where women in labor present to health centers before being referred to district hospitals for surgical care.

**Methods:**

this study included all adult women delivering via c-section between April 2017 and March 2018 in Kirehe District, Rwanda. We assessed the association between travel times and village-level c-section rates.

**Results:**

the estimated travel time from home-to-health center was 26 minutes (IQR: 13, 41) and from health center-to-hospital was 43 minutes (IQR: 2, 59). There was no significant association between travel time from home-to-health center and c-section rates (RR=1.01, p=0.42), but the association was significant for health center-to-hospital travel times (RR=0.96, p=0.01); for every 15-minute increase in travel time, there was a 4% decrease in c-sections for a health center catchment area.

**Conclusion:**

in the context of decentralized health services, minimizing health center to hospital referral barriers is of utmost importance for improving c-section access in rural sub-Saharan Africa.

## Introduction

Among women of childbearing age, pregnancy and delivery complications are the leading cause of death globally [1]. Each year, over 300,000 women die worldwide from complications associated with pregnancy and childbirth [2]. Of these deaths, 99% occur in low- and middle-income countries, of which over 60% occur in sub-Saharan Africa (SSA) [2]. More than 50% of maternal deaths are due to hemorrhage, sepsis, unsafe abortions, eclampsia, and obstructed labor [3]. Caesarean sections (c-sections) can have life-saving implications for the mother and/or her infant when pregnancy related complications such as placenta previa, or labor related complications such as prolapsed umbilical cord, or fetal distress. In 2015, an estimated 29.7 million c-sections - approximately 21% of all births globally - were performed[4]; however, the c-section rate in Eastern and Southern Africa stands at only 6.2% [4], and low c-section access is a contributing factor to the high maternal mortality rate of 546 per 100 000 live births [5]. Challenges including the limited human and financial resources and sub-optimal access to quality care limit the timely access to high-quality c-sections in SSA.

Many national health systems in Africa use decentralized care structures to provide much as close as possible to an individual´s home. In Rwanda, as in much of Africa, nurse-led peripheral health centers service most routine deliveries, while c-sections are often performed at district hospitals [6]. A woman first seeks care at her peripheral health center, where she is assessed and, if risks are identified, referred to the district hospital. However, the time to get to the health center, receive a referral, and then travel to the district hospital is critical and these delays can lead to an increased risk of neonatal and maternal mortality [3,7]. Rwanda has largely achieved the targets of decentralizing health care services, with health centers found located within one and a half hours by foot for all residents [8]. As of 2015, 90.7% women delivered in a facility and maternal mortality decreased from 750 to 210 per 100,000 live births in the previous decade [9]. Nationally, 13% of deliveries are via c-section, but access varies by region. However, in each region, the question remains whether in the context of decentralization, c-section access varies by proximity to health centers or hospitals. In this study, we examined the relationship between travel time through the referral system and c-section rates across Kirehe District, located in Rwanda´s Eastern province. Understanding how distance and referral steps are associated with c-section rates can inform future health systems planning and improve maternal and neonatal outcomes in Kirehe and other similar rural settings in SSA.

## Methods

**Study setting:** this study used data collected between April 1, 2017 to March 30, 2018 as part of a larger study on surgical site infections after c-section at Kirehe District Hospital (KDH) [10]. KDH is a district hospital servicing a catchment of over 360,000 people. Each year, KDH performs about 1400 surgical operations, the majority of which are c-sections [11]. In 2015, although 11% of all deliveries occurred in health facilities, Kirehe District reported that only 7.8% of deliveries occurred via c-section, well below Rwanda´s national average of 13% [9]. Kirehe District has 16 peripheral health centers plus two additional health centers from the neighboring Mahama refugee camp; each village is assigned a health center for primary health care. In Kirehe District, typically, a laboring woman first presents to a health center for delivery but will be referred to KDH if deemed necessary. An ambulance is the most common method to get from health center to hospital, but women may take private or public transportation if no ambulance is available. Kirehe´s population is largely rural and poor, with 90% of population involved in agriculture or livestock and only 56.2% of females having completed primary school [12].

### Data sources

**Geographic and population data:** we obtained village-level boundaries from the Database of Global Administrative Areas. Data on population per village, health center locations were obtained from Kirehe District records.

**Data on women undergoing c-section at KDH:** in the parent study, women who were at least 18 years old were prospectively enrolled after their c-section but prior to discharge. At the time of enrollment, study data officers collected data on demographics, health center visited, travel time and costs, and clinical history. In this study, we extracted data on the patient´s sector (the largest administrative unit under Kirehe District, with 12 sectors in the district), cell (the second largest administrative unit, with 60 cells in Kirehe District), and village (the smallest administrative unit, with 612 villages in Kirehe District). We compared the data against an official list of administrative areas obtained from Kirehe District records. Where there was no exact match, the Damerau-Levenshtein (DL) algorithm [13], with a limit of two edits, was used to find the best match between the reported village and a village from the list. For all included patients, 72% were exact matches to the official list, 25% were matched using the DL algorithm, 2.8% were matched manually, and two cases (0.2% of observations) were excluded as no match could be determined.

**Data on travel times from village to health center to KDH:** all referenced travel times in this paper were calculated using AccessMod version 5.0 [14]. The AccessMod calculation uses a least-cost path algorithm, taking roads, rivers and travel speeds into account [14]. We preferred AccessMod over the empirical travel times given by respondents because we can calculate travel time estimates for all villages, including 139 (23%) of the 612 villages that had no resident delivering via c-section during the study period and therefore did not have patient-reported travel time available. In areas where transportation is available, we assumed patients first walked to the nearest road and then took the motorized vehicle immediately available. We have previously demonstrated a strong correlation between AccessMod modeled travel times and patient reported travel times for travel from home-to-health center and health center-to-hospital [15].

**Statistical analyses:** the primary study outcome is the number of reported c-sections per population at the village-level. Crude c-section rates were calculated at the health center catchment (n=16) and village-level by dividing the number of recorded c-sections by the population size. Catchments are drawn with the goal of improving accessibility for patients, and therefore do not follow administrative sectors (n=12). To improve variance stability, we transformed village c-section rates to standardized incidence ratios (SIRs), by dividing village c-section rates by the overall c-section rate of the Kirehe District. For the map representations, village-level rates were smoothed using inverse distance weighting. We used a negative binomial regression model to assess the association between travel times from home-to-health center and health center-to-hospital and the village-level c-section incidence rates. Times were scaled to represent 15-minute increments. We used the Moran´s I Index on model residuals to determine if spatial dependencies were present. We reported coefficients, standard errors, risk ratios (RRs), 95% CIs and p-values for the results. Statistical significance was assessed at α =0.05 significance level. From the parent study, 14% of patients reported attending a health center other than their village´s assigned health center prior to traveling to KDH. Therefore, we conducted a sensitivity analysis to evaluate the relationship of travel time using a village´s most commonly reported health center, rather than its assigned health center, to address the 14% discrepancy between reported and assigned health centers. The population size of the catchment area was recalculated based on the reported health center. Data management and analyses were performed using R version 3. Mapping of figures and spatial analyses were performed using ArcGIS version 10.6.1.

**Ethics:** study participants provided a written informed consent prior to enrollment. Study data were entered directly into a REDCap database using password-protected, encrypted study tablets. A separate password-protected record was created linking patient identifying information and study IDs, which was destroyed at the end of data validation. This study obtained ethical approvals from the Rwanda National Ethics Committee (Kigali, Rwanda, No. 848/RNEC/2016) and Partners Human Research Committee (Boston, USA, No. 2016P001943/MGH). Prior to the start of data collection, the parent study was approved by the Rwandan Ministry of Health.

## Results

**Figure 1:**
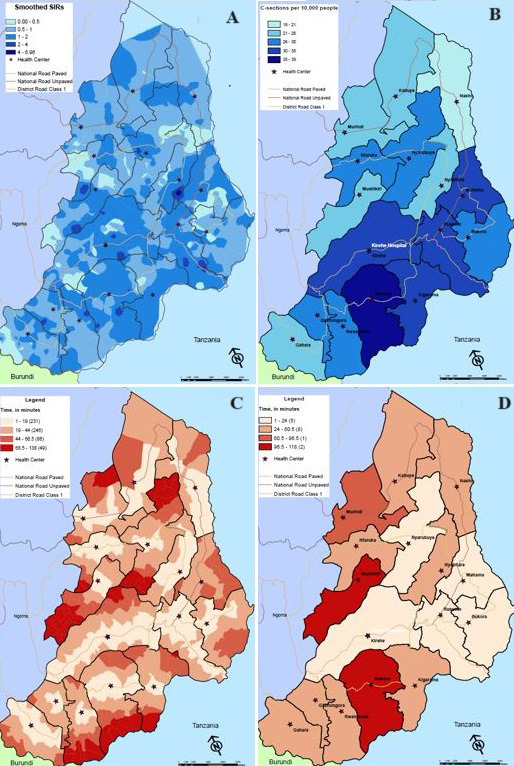
C-section rates and travel times, by village and health center catchment area. Inverse distance weighting smoothed c-section rates (A), scaled c-section rates by catchment-area (B), travel time from home-to-health center, in minutes (C), and travel time from health center-to-hospital (D), in minute

A total of 191 patients were excluded because they resided outside of Kirehe district or in Mahama Refugee Camp or, for two patients for whom no location data was available. This analysis covers 1,058 cases located in 612 villages. The median number of c-sections was 1 (interquartile range (IQR): 1,2) and in 139 (23%) village, no c-section was reported during the study window (Table 1). Among 473 villages that reported c-sections, women most commonly reported using public transportation when traveling from home-to-health center (n=326, 69%), followed by walking (n=88; 19%), and private transportation (n=5, 1%). The estimated travel time from home-to-health center was 26 minutes (IQR: 13, 41 minutes) (Table 1). Village-level smoothed SIRs and travel times from home-to-health center are shown in Figure 1A, and Figure 1C, respectively.

**Table 1:** village and health center catchment-level summaries of c-section numbers, rates and travel times

Variables	Median [IQR] unless specified
Village-level	n = 612
Number of c-sections recorded	1 [1,2]
C-section rates, per 10,000 people	24 [12, 42]
SIR*	0.83 [0.41, 1.42]
Population	555 [431,709]
Travel time: home-to-health center, minutes	26[13,41]
Health center catchment-level	n = 16
Number of c-sections recorded	59 [41, 76]
C-sections per 10,000 people*	28[25, 31]
Population	20641 [14019,25590]
Travel time: health center-to-hospital, minutes	43 [27, 59]
Health centers with a designated ambulance, n (%)	6 (38%)

* SIR (standardized incidence ratio)= (c-sections at Kirehe District Hospital with patient from village/village population)/(total c-sections at Kirehe District Hospital/ district population)

**Figure 2:**
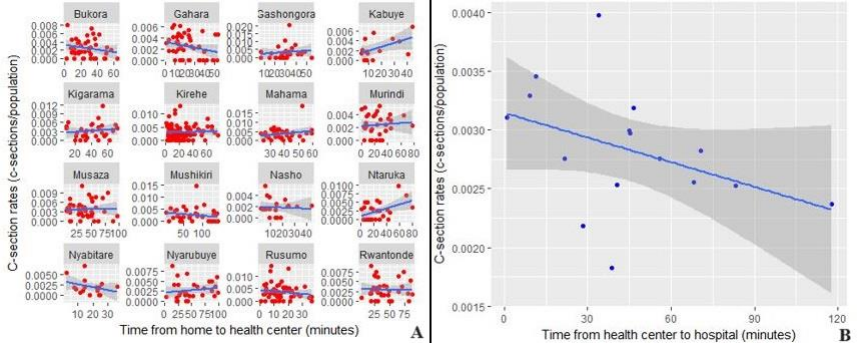
C-section rates vs. time from home-to-health center, grouped by health center catchment level (A) and from health center-to-hospital (B)

The median number of c-sections referred from each health center over the year was 59 (IQR: 41, 76). Health center-level c-section rates are represented in Figure 1B. At Kirehe Health Center -located on the same campus as KDH - nearly all 200 (19%) women who first presented to this health center walked to the hospital. Among the remaining 858 women, 576 (67%) traveled from the health center to the hospital via an ambulance, with six of the 16 health centers (38%) having an ambulance based on site (Table 1). The median estimated travel time from health center-to-hospital was 43 minutes (IQR: 2, 59 minutes). Travel times from health center-to-hospital are presented in Figure 1D, and Figure 2 shows the relationship between the travel times and c-section rates.

There was no significant association between travel time from home-to-health center and village-level c-section rates (RR=1.01, 95% CI: 0.97, 1.07, p=0.42) (Table 2). However, there was a significant association between travel time from health center-to-hospital and village-level c-section rates (RR=0.96, 95% CI: 0.92, 0.99, p=0.01). Specifically, for every 15-minute increment increase in travel time, there is an estimated 4% decrease in c-sections for a health center catchment area. Results of the Moran´s I Index of model residuals indicated no spatial autocorrelation (p =0.35), and so this model was not adjusted further for spatial dependency. The results remained consistent in the sensitivity analysis (association of home-to-health center travel times, RR=1.03, 95% CI: 0.99, 1.08, p=0.11; association of health center-to-hospital travel times, RR=0.94, 95% CI: 0.91, 0.98, p<0.01).

**Table 2:** results of the model examining the relationship of village-level c-section rates and travel time

Parameter	RR [95% CI]	p-value	Beta (SE)
Time from home-to-health center*	1.01 [0.97, 1.07]	0.42	0.019 (0.023)
Time from health center-to-hospital*	0.96 [0.92, 0.99]	0.01	-0.045 (0.018)

* 1-unit change is equal to a difference in 15 minutes of travel time

## Discussion

In this analysis, we found that village c-section rates in rural Rwanda are associated with travel time to the district hospital. Specifically, the further a village´s assigned health center was from the district hospital, the lower the rate of c-section in that village. In addition to decreased rates of c-section, this distance has also been found to be associated with poorer neonatal outcomes post-cesarean delivery [16,17]. One plausible explanation for this result is Kirehe´s emergency referral network to transport patients from the health centers to the hospital. Previous studies have reported ineffective referral systems, specifically in coordination between different levels of the referral system, as a barrier to care in rural settings [18]. Increasing investments in road infrastructure or resources, such as additional ambulances, could decrease the impact of transportation times, which have been identified as barriers to care in rural regions [19]. Distance and travel times from health center-to-hospital could also be masking other issues, such as costs and quality of facility, which have also been identified as obstacles for care [20-22].

Surprisingly, the travel time from home-to-health center was not associated with c-section rates. The Rwanda MOH has been directing efforts towards decentralization of health centers since 2001, aiming to have health centers within one and a half hours by foot for all residents [8]. Rwanda´s focus on decentralization, along with improving effectiveness and enhancing responsiveness of delivery of services, has resulted in improvements in health coverage including antenatal care which helps women in pregnancy and delivery planning, and rates of facility deliveries by a skilled health worker [23].In Rwanda´s 2015 Demographic Health Survey, 90.7% of deliveries reportedly occurred in a facility [9,24], well above the 2014 sub-Saharan Africa regional median of 57% [25]. We are currently supporting processes to better plan for c-section delivery when medically indicated during pregnancy, which would avoid the need for referral during labor and barriers to access [26]. Of note, we have previously found that the travel costs from home-to-health center are associated with higher rates of surgical site infections post c-section in Kirehe district [27], suggesting that these distances may hinder post-c-section follow-up care even if they are not a contributor to access to c-section itself.

This study had several limitations. We calculated the denominator using the village population size rather than the number of village births as this data was unavailable. This requires the assumption that the birth rates are homogeneous across the district; while this is untestable, we believe this is a reasonable assumption given the relative homogeneity of the district on other related demographics. Additionally, we focused only on travel time, but other factors could be influencing c-section rates. Factors such as seasonality, wait times at health centers, and health center quality could be correlated with distance and should be studied in the future.

## Conclusion

The c-section rate in Kirehe district is lower than the national average and there is further variability in accessibility across the district. The lack of effect of travel time from home-to-health center may confirm the value of healthcare decentralization overall. However, since c-sections only occur at the hospital, to remove the effect of travel time from health center-to-hospital on village-level c-section rates, measures to strengthen the referral system between the health centers and district hospital should be implemented and then studied for impact.

### What is known about this topic

- *Distance to health facilities is inversely related to access to health care, including surgery;*
- *Cesarean delivery rates in sub-Saharan Africa are low and variable;*
- *What is unknown is how distance affects cesarean access*.

### What this study adds

- *Travel time from health center to hospitals are associated with c-section rates, with a 15-minute increase in this travel time correlating to a 4% decrease in c-sections;*
- *Travel time from home to health center was not associated with c-section rates;*
- *Better intrafacility referral systemsare needed to ensure equitable c-section access*.
